# Comparing fluorodeoxyglucose positron emission tomography with computed tomography in staging for nodal and distant metastasis in urothelial/bladder cancer

**DOI:** 10.1002/bco2.304

**Published:** 2024-02-20

**Authors:** Mohammed Al‐Zubaidi, Katherine Ong, Pravin Viswambaram, Haider Bangash, Glenn Boardman, Steve P. McCombie, Oliver Oey, Nicole Swarbrick, Andrew Redfern, Jeremy Ong, Richard Gauci, Ronny Low, Dickon Hayne

**Affiliations:** ^1^ Department of Urology Fiona Stanley Hospital Murdoch Australia; ^2^ UWA Medical School University of Western Australia Crawley Australia; ^3^ Research Support and Development Unit Fiona Stanley Hospital Murdoch Australia; ^4^ Pathwest Fiona Stanley Hospital Murdoch Australia; ^5^ Department of Medical Oncology Fiona Stanley Hospital Murdoch Australia; ^6^ Department of Nuclear Medicine Fiona Stanley Hospital Murdoch Australia; ^7^ Department of Radiology Fiona Stanley Hospital Murdoch Australia

**Keywords:** bladder cancer, CT scan, FDG‐PET, metastasis, neoadjuvant chemotherapy, radical cystectomy, urothelial carcinoma

## Abstract

**Objectives:**

We aim to assess the clinical value of ^18^F‐fluorodeoxyglucose positron (^18^F‐FDG‐PET) scan in detecting nodal and distant metastasis compared with computed tomography (CT) scan in patients with urothelial carcinoma or bladder cancer, aiming to improve staging accuracy and thereby better prognosticate and determine therapy.

**Methods:**

A retrospective review of 75 patients with invasive bladder cancer (≥T1) who were staged with both CT and ^18^F‐FDG‐PET within an 8‐week interval was performed for the period between 2015 and 2020. Seventy‐two per cent (54/75) had formal pelvic lymph node (LN) dissection or biopsy of lesions suspicious for metastases. FDG‐PET definitions for positive sites were assessed depending on SUV Max (nodes with SUVmax >4 at any size, SUV > 2 for lymph nodes >8 mm, or any SUV if the lymph node was >10 mm on axial images). For CT scanning, enlarged LN by RECIST 1.1 criteria (>10 mm) as well as qualitative findings suggesting metastasis were considered positive. The analysis was based on the comparison of CT and ^18^F‐FDG‐PET findings to histopathology results from LN dissection or biopsies.

**Results:**

Sensitivity, specificity, positive predictive values (PPV) and negative predictive value (NPV) of CT versus FDG‐PET for detecting metastasis, in patients who underwent pelvic LN dissection or biopsy of lesions suspicious of metastases, were 46.6% (95% CI: 21%–70%) versus 60% (95% CI: 32%–84%), 100% (95% CI: 91%–100%) versus 83.78% (95% CI: 69%–94%), 100% (95% CI: 63%–100%) versus 60% (95% CI: 32%–84%), and 82.2% (95% CI: 68%–92%) versus 83.78% (95% CI: 69%–94%), respectively. 7/75 (9.3%) patients avoided cystectomy due to ^18^F‐FDG‐PET features of metastases that were not detected by CT.

**Conclusion:**

FDG‐PET may be more sensitive than CT for metastases in the staging of bladder cancer, which resulted in significant avoidance of aggressive local management in cases with occult metastasis.

## 1 INTRODUCTION

Urothelial carcinoma (UC) of the bladder is the most common malignancy of the urinary tract with the highest lifetime treatment cost per patient of all cancers.^1^ At the time of initial diagnosis, approximately 70%–75% are non‐muscle invasive, 20%–25% are muscle invasive with 5%–10% of these being metastatic.^2^ The most frequent sites for metastatic disease are pelvic lymph nodes, most commonly obturator (74%) and external iliac nodes (65%).^2^ Less commonly, pre‐sacral nodes (25%), common iliac nodes (20%) and para‐vesical nodes (16%) may be involved.^2^ Distant metastatic sites for UC include the lung, liver, bone, adrenals, skin and, more rarely, the central nervous system. The 5‐year survival for non‐metastatic muscle‐invasive UC is approximately 70% reducing to 6%–33% in metastatic disease.^3^ Accurate staging of muscle‐invasive bladder UC is crucial in the decision‐making process and selection of optimal treatment modality. Under‐staging potentially exposes patients to overtreatment and the risks of undergoing unnecessary radical surgery, which is associated with significant morbidity and complications. Over‐staging risks denying patients potentially curative treatment with consequent progression of the disease and worse overall survival.

Radical cystectomy (± NAC) with pelvic lymph node dissection is considered the gold standard treatment for localised muscle‐invasive bladder cancer. Conventionally, as supported by international guidelines, staging to assess for nodal and distant metastatic disease in muscle‐invasive UC is performed using the CT scan.^4^, ^5^, ^6^ ^18^F‐FDG‐PET/CT is a combined imaging modality that involves structural assessment on CT combined with assessment of glycolysis, a feature of many malignancies.^7^, ^8^, ^9^, ^10^, ^11^ The current study was designed to determine whether ^18^F‐FDG‐PET has better urothelial cancer staging yields than conventional CT of the chest, abdomen and pelvis.

## 2 METHODS

### 2.1 Patients

We retrospectively reviewed 75 patients with invasive bladder cancer (≥T1) between 2015 and 2020 at a single tertiary centre. Patient selection was based on either high‐grade histopathology results from the time of endoscopic resection or imaging‐guided biopsy of a suspicious metastatic lesion. All patients underwent a CT scan of the chest, abdomen and pelvis, and an ^18^F‐FDG‐PET scan, with both scans having been performed within an 8‐week interval and prior to neoadjuvant chemotherapy (NAC) (if applicable). No patient had staging scans using multiparametric magnetic resonance imaging (MRI). Patients were excluded from the study if these scans were not performed within an 8‐week interval.

**FIGURE 1:**
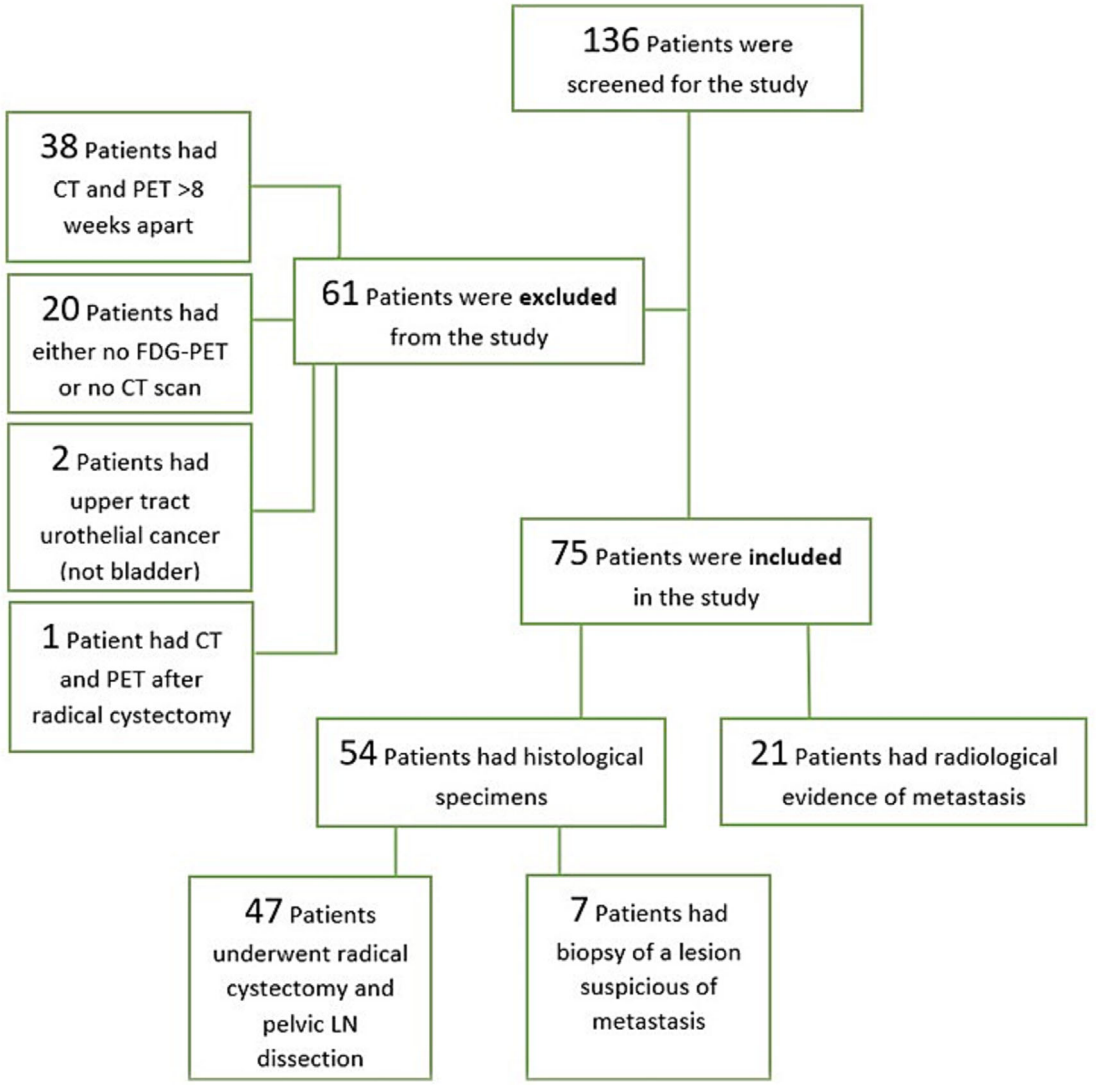
Consort diagram of patients screened in the study.

Fifty‐four patients had either a formal pelvic LN dissection, performed by the same surgeon in a single facility, or a biopsy of a lesion suspicious for metastasis, performed by an interventional radiologist at the same centre (Figure 1). The surgeon performing the pelvic LN dissection was not blinded to the FDG‐PET scan results; however, a standard complete pelvic LN dissection template was used (obturator, internal iliac, external iliac and common iliac nodes up to the crossing of the ureters). Histopathological findings from surgical specimens or image‐guided biopsies were considered the gold standard in comparison with the imaging modalities. Histopathological examination was done by the same pathology centre at the same tertiary hospital.

### 2.2 ^18^F‐FDG‐PET imaging

Patient preparation for whole‐body PET included fasting for at least 6 h prior to FDG injection. A weight and height‐based dose of ^18^F‐FDG was administered intravenously after which the patient rested quietly for 60 min. Whole‐body images were acquired, with an effective field of view of the vertex to thighs, typically with the arms up.

### 2.3 Data analysis

The CT and ^18^F‐FDG‐PET/CT studies were independently re‐reviewed by an experienced radiologist and a dual‐trained radiologist and nuclear medicine specialist, blinded to reports and the other staging modality images. Enlarged LN by RECIST criteria 1.1 (>10 mm) and other qualitative findings suggesting metastasis were considered positive in CT scan. For FDG‐PET, nodes or lesions with SUVmax >4 at any size, SUV > 2 for lymph nodes >8 mm, or any SUV if the lymph node was >10 mm were considered positive on ^18^F‐FDG‐PET/CT scan. Focal extra‐nodal abnormalities were considered positive for metastasis based on qualitative visual assessment; no SUV cut‐off was used in line with clinical practice. Lesions suspicious for metastasis were considered positive upon biopsy confirmation or after discussion at the uro‐oncology multidisciplinary meeting.

### 2.4 Correlation between imaging and histopathology results

Patients who were deemed to have positive pelvic nodes on CT or ^18^F‐FDG‐PET that corresponded, in location and lymph node group, to the histologically positive nodes (on biopsy or lymph node dissection) were considered as true positive. Patients who had positive nodes in either imaging modality but were negative on histopathology of the same lymph node group were labelled as false positive for nodal staging. The same criteria were utilised for distant lesions suspicious for metastases on either imaging modality when compared with histopathology results from a biopsy of those sites. Patients who underwent NAC were included in our study.

### 2.5 Statistical analysis

The sensitivity, specificity, positive predictive value and negative predictive value of both CT and FDG‐PET scans were calculated using the standard definition. The sensitivity and specificity of FDG‐PET versus CT were compared using McNemar's test.

### 2.6 Ethics

This study was approved by the South Metropolitan Health Service research governance body (GEKO approval number 37901).

## 3 RESULTS

**FIGURE 2:**
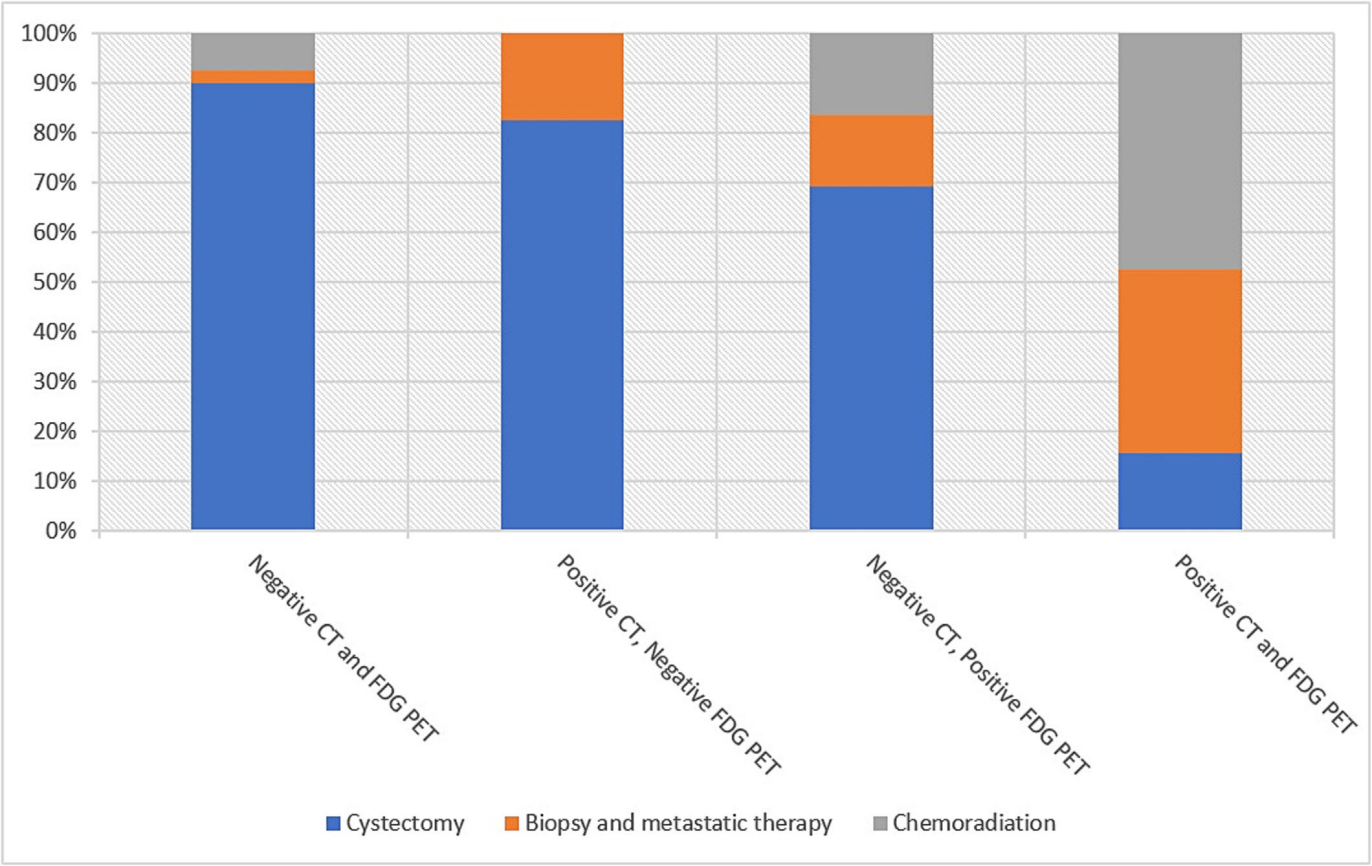
Correlation between staging imaging results and subsequent management plan.

One hundred thirty‐six patients with high‐grade urothelial carcinoma or bladder cancer were initially identified, of whom 75 patients were eligible to be included in the study (Figure 1). Fifty‐four patients (72%) had histopathology available from pelvic lymph node dissection or biopsy. Following initial staging scans with CT and FDG‐PET scans, 87% (47/54) of patients underwent radical cystectomy and pelvic lymph node dissection (Table 1) with a median node count of 17 (0–52 nodes),^12^ and 13% (7/54) had radiologically guided biopsy for histological confirmation of lesions suspicious for metastasis (Table 2). The remaining 21 patients who had histologically confirmed muscle‐invasive urothelial carcinoma from endoscopic resection with radiological evidence of metastatic disease on CT or PET scans were deemed not to require histological confirmation of metastasis as per the consensus of the uro‐oncology multidisciplinary meeting. These patients were treated with either chemotherapy, chemoradiation or radiotherapy. The above is summarised in Figure 2.

**TABLE 1:** Radical cystectomy patients' demographics.

Demographics	Value
Gender	
Male (*n*)	39
Female (*n*)	8
Age	
Range (years)	44–89
Median (years)	70
Radical cystectomy (*n*)	47
Indications for surgery	
Muscle invasive bladder cancer (at least T2) (*n*)	32
High‐grade NMIBC unresponsive to BCG (*n*)	7
High‐grade NMIBC without previous BCG (*n*)	6
Adenocarcinoma (*n*)	2
Neoadjuvant chemotherapy (*n*)	12

**TABLE 2:** Biopsy sites.

Location of histologically confirmed metastasis (Stage)	Number of patients
Left second rib (M1b)	1
Liver (M1b)	1
Adrenal gland (M1b)	1
Bone (Femur) (M1b)	2
Ileocecum segment of bowel (M1b)^a^	1
Common iliac node (N3)	1

^a^ Patient had a right pelvic lesion invading bowel, histology confirmed metastatic urothelial carcinoma upon bowel resection.

Twenty‐two per cent (12/54) of patients undergoing radical cystectomy received NAC between their staging and surgery. All these patients had negative staging on both CT and PET staging, and only one of these patients had lymph node metastasis identified on final pathology.

Sensitivity, specificity, PPV and NPV of CT for detecting pelvic LN metastases were 46.6% (95% CI: 21%–70%), 100% (95% CI: 91%–100%) 100% (95% CI: 63%–100%) and 82.2% (95% CI: 68%–92%), respectively. Sensitivity, specificity, PPV and NPV of ^18^F‐FDG‐PET for detecting pelvic LN metastases were 60% (95% CI: 32%–84%), 83.78% (95% CI: 69%–94%), 60% (95% CI: 32%–84%), and 83.78% (95% CI: 69%–94%), respectively (Table 3). Overall, FDG‐PET was not significantly more diagnostic than CT for pelvic LN metastases (*p* = 0.4497).

**TABLE 3:** Sensitivity, specificity, PPV and NPV for both imaging modalities.

	CT scan (%)	^18^FDG PET/CT scan (%)
Sensitivity	46.6	60
Specificity	100	83.78
Positive predictive value (PPV)	100	60
Negative predictive value (NPV)	82.2	83.78

**FIGURE 3:**
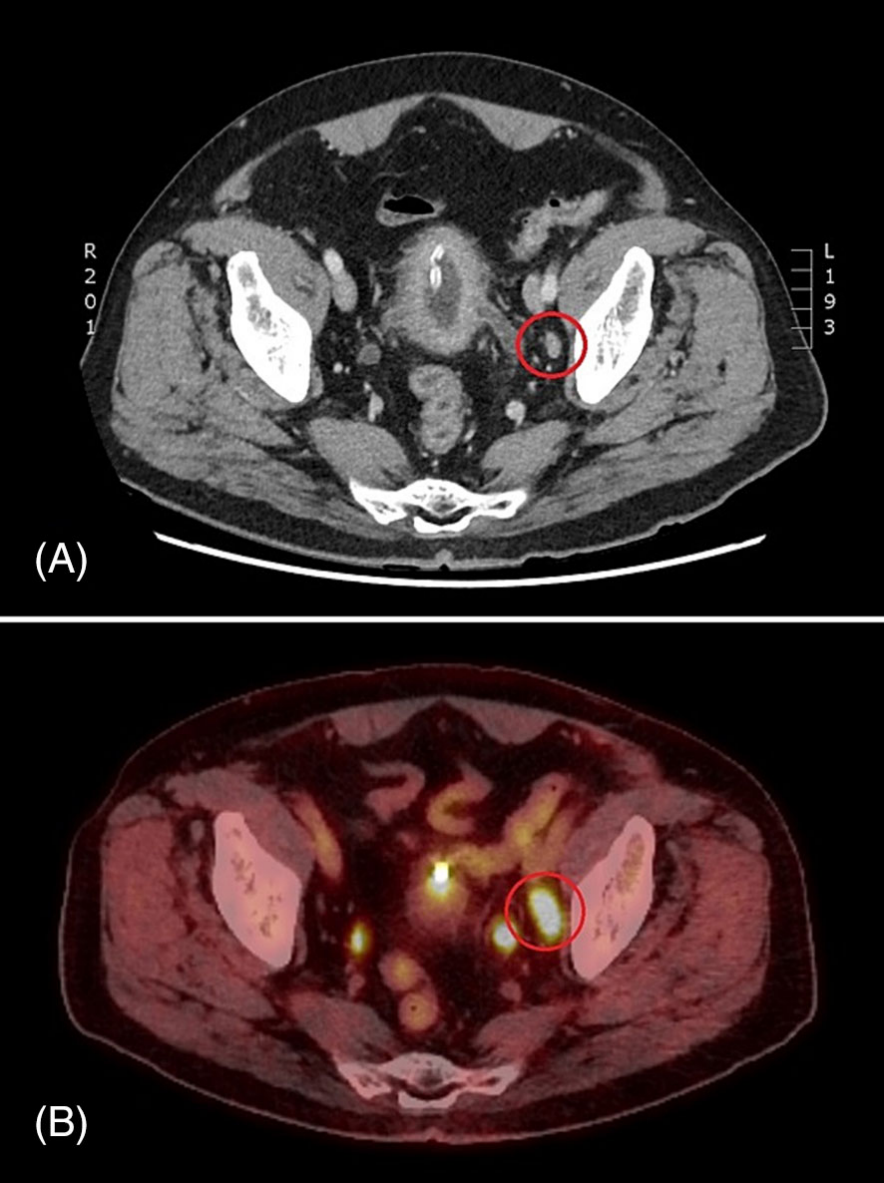
CT and FDG PET of the same patient (<8 weeks apart) showing (A) a negative CT as per RECIST criteria [small left external iliac lymph node in CT (8 mm)] and (B) a positive FDG PET showing same LN with SUV max 9.9 (SUVmax >4).

Twenty‐five patients had identified metastatic disease in ^18^F‐FDG‐PET, compared with 17 patients with metastasis visible on CT. Staging with ^18^F‐FDG‐PET identified metastatic disease in 10.6% (8/75) of patients where metastases were occult on CT, whereas no patient had metastases visible on CT that were occult to ^18^F‐FDG‐PET. For instance, a patient had a positive regional LN on PET, which was not considered positive by CT criteria. Therefore, radical cystectomy with a standard PLND template was performed (Figure 3).

This study showed that 9.3% (7/75) of patients avoided inappropriate radical local treatment, either with cystectomy or radiotherapy, due to the ^18^F‐FDG‐PET diagnosis of metastases based on SUV criteria. Six patients had disease progression upon further follow‐up with interval scans within the first few months of commencing the multidisciplinary treatment. The seventh patient had complete radiological resolution after four cycles of carboplatin/gemcitabine. However, a radiological recurrence of FDG avid metastases was detected 2 years later mandating the commencement of immunotherapy with subsequent further progression of the disease.

## 4 DISCUSSION

Accurate staging of muscle‐invasive bladder cancer is vital in defining optimum patient management. Radical local treatments, such as radical cystectomy, can be highly effective in treating organ‐confined disease^13^ but are ineffective treatments with a high rate of unnecessary complications, for instance, bowel obstruction, urinary leak and metabolic complications, if utilised in the presence of metastatic disease.

CT criteria for abnormal lymph nodes are predominantly based on size, such that CT staging is affected by limited sensitivity for small, involved nodes. In addition, benign lymph nodes can theoretically be enlarged due to infection or recent regional procedures although false positives are also possible for FDG‐PET in these scenarios. Consequently, specificity in evaluating enlarged nodes is occasionally compromised in both investigation modalities.^14^

^18^F‐FDG‐PET/CT is a hybrid imaging technique that facilitates both structural assessment of organs, such as lymph nodes, and evaluating glycolytic activity, a feature common to many malignancies.^15^ It is therefore widely used in the initial staging and treatment response assessment of many malignancies, particularly to define the suitability of radical local treatment. While urinary excretion of FDG limits assessment of local urinary tract lesions on PET, it is possible to augment imaging protocols with diuretics to permit urinary tract lesion assessment.^16^, ^17^, ^18^, ^19^, ^20^ Early phase evaluation of molecular targets in urothelial malignancy, such as carbonic anhydrase IX (radiolabelled girentuximab), allowing reagents that are hepatically cleared, is underway.^21^

There have been limited reports comparing preoperative staging of bladder cancer with ^18^F‐FDG‐PET and conventional CT. In a cohort of 300 patients, FDG‐PET was more accurate than conventional CT staging alone for preoperative LN staging with a sensitivity of 40.3% compared with 13.4% for CT.^22^ In our study, the sensitivity of both modalities was higher, although the sensitivity of CT remained relatively low, raising a major concern in relying solely on this for staging. In contrast, CT showed a specificity of 100% meaning that there were no reported reactive enlarged (≥10 mm) lymph nodes on CT scan with a negative disease on histopathology. Specificity was 83% for FDG‐PET indicating that false positives occurred and suggesting that confirmatory biopsy should still be considered as an adjunct to a positive FDG‐PET scan. Most importantly, 9.3% of patients avoided cystectomy due to ^18^F‐FDG‐PET features of metastases that were not detected by CT.

Clinical staging errors utilising CT without FDG‐PET in patients with muscle‐invasive bladder cancer have been reported to occur in up to 40% of patients, thereby conferring a high risk of inappropriate treatment implementation.^23^ ^18^F‐FDG‐PET may improve the selection of the most appropriate treatment option due to improved staging accuracy. Furthermore, it may allow more accurate treatment planning, for example, through targeting of involved nodes with LN dissection or radiotherapy.

The additional value of imaging with PET has become increasingly recognised for other primary tumour sites. For instance, PSMA PET provided superior accuracy to CT and bone scan combined in prostate cancer staging (92% vs. 65%, *p* < 0.001), prompting it to recently be approved in Australia for routine staging.^24^

There are several limitations to consider in our study. It is a retrospective review of a single centre with a relatively small study population. Although patients who underwent NAC were included in our study, the effect of NAC was not formally assessed. It may have potentially down‐staged disease in the pelvic nodes with a subsequent risk of overestimating the specificity of CT and PET scans in these patients. The location of the involved lymph nodes was not accurately reported on the pelvic lymph node dissection as specimens were sent as nodal groups. This analysis is also based on the assumption that all pathological pelvic lymph nodes were removed at the time of cystectomy, although the reported high lymph node yields are reassuring regarding the quality of the dissection. Finally, not all patients treated as metastatic disease had histological confirmation of metastasis. For instance, 9.3% of patients in this study were treated for metastatic disease relying on radiological evidence of metastasis only.

## 5 CONCLUSION

FDG‐PET detected metastatic disease missed by conventional CT in approximately 10% of patients resulting in significant management changes. ^18^F‐FDG‐PET may thus provide additional diagnostic reliability over CT in patients with muscle‐invasive bladder cancer. It may increase the sensitivity for the detection of nodal or metastatic disease, potentially at the expense of reduced specificity. Further research comparing the diagnostic yield of ^18^F‐FDG‐PET and CT in detecting nodal and distant metastasis in bladder cancer may be warranted.

## AUTHOR CONTRIBUTIONS

**Mohammed Al‐Zubaidi:** Data analysis and manuscript writing. **Katherine Ong:** Manuscript editing. **Pravin Viswambaram:** Data collection. **Haider Bangash:** Manuscript editing. **Glenn Booradman:** Statistical support. **Steve McCombie:** Manuscript editing and supervision. **Oliver Oey:** Data collection. **Nicole Swarbrick:** Manuscript editing and histopathology support. **Andrew Redfern:** Manuscript editing. **Jeremy Ong:** Manuscript editing and nuclear medicine/radiology support. **Richard Gauci:** Manuscript editing and nuclear medicine support. **Ronny Low:** Manuscript editing and Radiology support. **Dickon Hayne:** Manuscript review, editing and main supervisor.

## CONFLICT OF INTEREST STATEMENT

None.
